# Deferoxamine attenuates visual impairment in retinal ischemia‒reperfusion via inhibiting ferroptosis

**DOI:** 10.1038/s41598-023-46104-0

**Published:** 2023-11-17

**Authors:** Xiaoxuan Wang, Mingran Li, Ke Diao, Yan Wang, Hong Chen, Ziqi Zhao, Yuan Li, Xin Jia, Hao Wang, Fangyuan Zheng, Zihan Xia, Longhui Han, Minglian Zhang

**Affiliations:** 1 Department of Ophthalmology, Hebei Medical University, Shijiazhuang, 050017 Hebei China; 2 Hebei Eye Hospital, Hebei Provincial Key Laboratory of Ophthalmology, Hebei Provincial Clinical Research Center for Eye Diseases, Xingtai, 054000 Hebei China

**Keywords:** Diseases, Medical research

## Abstract

Retinal ischemia‒reperfusion (I/R) injury can cause significant damage to human retinal neurons, greatly compromising their functions. Existing interventions have been proven to have little effect. Ferroptosis is a newly discovered type of programmed cell death that has been found to be involved in the process of ischemia‒reperfusion in multiple organs throughout the body. Studies have shown that it is also present in retinal ischemia‒reperfusion injury. A rat model of retinal ischemia‒reperfusion injury was constructed and treated with deferoxamine. In this study, we found the accumulation of Fe^2+^, reactive oxygen species (ROS), malondialdehyde (MDA), and the consumption of glutathione (GSH) via ELISA testing; increased expression of transferrin; and decreased expression of ferritin, SLC7A11, and GPX4 via Western blotting (WB) and real-time PCR testing. Structural signs of ferroptosis (mitochondrial shrinkage) were observed across multiple cell types, including retinal ganglion cells (RGCs), photoreceptor cells, and pigment epithelial cells. Changes in visual function were detected by F-VEP and ERG. The results showed that iron and oxidative stress were increased in the retinal ischemia‒reperfusion injury model, resulting in ferroptosis and tissue damage. Deferoxamine protects the structural and functional soundness of the retina by inhibiting ferroptosis through the simultaneous inhibition of hemochromatosis, the initiation of transferrin, and the degradation of ferritin and activating the antioxidant capacity of the System Xc-GSH-GPX4 pathway.

## Introduction

Retinal ischemia‒reperfusion injury is a basic pathogenic factor associated with multiple eye diseases, including retinal vascular occlusion and glaucoma^1–3^. Studies have also found the involvement of multiple types of cell death underlying the pathogenic processes, including necrosis, pyroptosis, and apoptosis^4,5^. However, recently, the research focus has turned toward ferroptosis, a newly discovered type of programmed cell death^6^. There is sufficient cumulative evidence that supports the involvement of ferroptosis in the pathogenesis and development of many diseases^6–9^, where iron overload in the cells generates reactive oxygen species (ROS) through Fenton reactions and thereby contributes to the oxidative damage of the cells^7,10–12^. Iron chelators such as deferoxamine, ferrostatin-1, and liproxstatin-1 may inhibit this type of cell death^13–15^.

Ferroptosis is an important factor underlying cell death caused by I/R injury^16,17^, especially in cells situated in the brain and intestine^17,18^. Galina Dvoriantchikova et al. identified at least four types of programmed cell death in retinal I/R injuries through RNA sequencing: necroptosis, pyroptosis, ferroptosis, and parapoptosis^19^. Although this study discovered genetic changes related to ferroptosis when I/R injuries occur and the correlation of the four cell death types through gene sequencing, it stopped short of any further investigations into the pathogenic mechanism of ferroptosis in retinal I/R injuries.

In one of the most recent studies, researchers found, by monitoring the levels of the key proteins involved in apoptosis, necrosis, and ferroptosis, that these three cell death types coexist in retinal I/R injuries and that the combined use of z-VAD-FMK (apoptosis inhibitor), necrostatin-1 (necrosis inhibitor), and ferrostatin-1 (ferroptosis inhibitor) can reduce the amount of retinal ganglion cell death after retinal I/R injuries^20^. Currently, studies on ferroptosis in retinal ischemia‒reperfusion mainly focus on RGCs. It has not been investigated whether ferroptosis also occurs in other cell types in retinal I/R injuries. In the management of retinal I/R injuries, ferrostatin-1 has been the most common ferroptosis inhibitor of choice^21^. It is an antioxidant that prevents membrane lipid damage and thereby inhibits cell death through reduction reactions^22^. However, an intervention that inhibits ferroptosis by regulating the iron level and key factors of iron metabolism is still absent on the market.

The present study found that in retinal I/R injuries, ferroptosis-inducing factors are upregulated and ferroptosis-inhibiting factors are downregulated, leading to the accumulation of Fe^2+^, ROS, and malondialdehyde (MDA), which consequentially induces ferroptosis in multiple cell types, including RGCs, photoreceptor cells, and retinal pigment epithelium cells. We found that the iron chelator deferoxamine could inhibit ferroptosis by targeting excessive iron accumulation in the retina after I/R injury, which could prevent the death of RGCs and the abnormal proliferation of Müller cells and thus protect the structure and function of the retina. These findings provide a promising strategy for the management of ischemic eye diseases.

## Materials and methods

### Animals and ethics statement

This animal experiment was approved by the Animal Experiment Ethics Committee of Hebei Medical University and conducted in compliance with the Association for Research in Vision and Ophthalmology Statement for the Use of Animals in Ophthalmic and Vision Research. The study used clean-grade male Sprague‒Dawley rats (Beijing Charles River Laboratories) weighing 200–250 g. All animals were reared in a 12-h light/dark cycle environment after purchase and fed sufficient food and water. All experimental procedures and inspections were conducted when the rats were under anesthesia: the rats were administered 10% chloral hydrate (4 mL/kg) via intraperitoneal injections and kept on a heat preservation pad to maintain their body temperature to avoid hypothermia-related deaths during the procedures.

### Establishment of the retinal ischemia‒reperfusion model

The retinal ischemia‒reperfusion models used in this study were established according to relevant literature^23^: the rats were placed under deep systemic anesthesia and received local anesthesia of the eyes using 0.5% oxybuprocaine eye drops. We used 0.5% chloramphenicol + dexamethasone eye drops to clean the conjunctival sacs and iodine solutions to disinfect the areas around the eyes. Then, the rat pupils were dilated with compound tropicamide eye drops. Then, the anterior chamber in the left eye was cannulated with a 30 G needle connected to a reservoir containing 0.9% NaCl. The reservoir was raised to a height of 1496 mm above the eye to elevate the IOP to 110 mmHg^23^. The rats’ eyeballs visibly hardened, and the bulbar conjunctiva turned pale. After 60 min, the blood supply to the retina was restored by gradually lowering the infusion vial to the level of the rat’s eyeballs to slowly reduce the intraocular pressure. Then, the infusion rod was turned off, and the needle in the anterior chamber was pulled out. The presence of reperfusion was confirmed by the orange‒red color of the retina, indicating that the blocked blood vessels reopened, by which point the building of the I/R model was complete^3^. Gatifloxacin ophthalmic gel was used locally during the operation to protect the cornea, and tobramycin + dexamethasone eye ointment was applied locally after the operation twice a day to prevent infection. The sham operation, which served as the control, was performed without elevating the IOP.

### Animal grouping and deferoxamine treatment

The rats were euthanized at 24 h, 48 h and 72 h after lesion induction. Then, the rats were allocated to 4 randomized groups: the control + saline group, the I/R + saline group, the control + deferoxamine group, and the I/R + deferoxamine group. Deferoxamine was prepared as a solution with normal saline. Rats in the deferoxamine treatment group were administered deferoxamine (100 mg/kg, MCE, HY- B0568) via intraperitoneal injections for the first time 1 h before anesthesia and then once every 12 h after they recovered from anesthesia; the rats in the saline group were intraperitoneally injected with the same amount of saline at the same time point. The rats were euthanized at 48 h after injury.

### Visual evoked potential (VEP)

The rats’ VEP data were recorded by the standardized protocol for rat visual evoked potential recording, where the rats were anesthetized intraperitoneally and their pupils dilated, and three electrodes were inserted under the skin of the occipital bone (active electrode), bregma (reference electrode) and the ears (ground electrode). When the preparation was completed, the VEP of both eyes of each rat was taken independently (i.e., monocular detection, with the eye not in examination covered in light-proof covers) via F-VEP (Germany Roland RETI-Port21). The P2 latency and N2-P2 amplitude were measured^23^.

### Electrophysiology (ERG)

The rats’ ERG was taken by the standardized protocol of rats. The rats were placed in a dark box for 24 h of dark adaptation and then transferred to a dark room where the rats were abdominally anesthetized under weak red light. After their pupils were properly dilated, corneal electrodes were placed on the corneas of both eyes, and the ground electrodes were subcutaneously inserted into the tail. The reference electrodes were inserted into the buccal mucosa bilaterally. F-ERG examination was performed (RETI-Port21, Roland, Germany), and the a and b wave amplitudes of the F-ERG in both eyes were recorded. F-ERG data from the left eyes of all rats were used for the statistical analysis^24^.

### Determination of iron content

The iron content in the retinal tissue was determined using an iron content assay (Nanjing Jiancheng Bioengineering, A039-2–1). The retinal sample was prepared as a homogenized solution with normal saline and then centrifuged at 2500 rpm for 10 min; the supernatant was taken for testing. Iron chromogenic reagents were added to the standard reference and the test samples. The materials were then mixed well, bathed in boiling water for 5 min, and cooled in running water before being centrifuged at 3500 rpm for 10 min. Then, 1.0 mL of the supernatant from each tube was taken to be measured for the optical density (OD) value at 520 nm with a spectrophotometer.

### Determination of ROS and glutathione (GSH) levels

The ROS and GSH levels were determined with ROS (Beijing Bai’aolai Technology Co., Ltd., HR8821) and GSH (Abcam, ab138881) assays. The retinal tissue was lysed and centrifuged at 13,000 × g and 4 °C for 10 min. The supernatant was taken for the assays. The samples were examined under a fluorescent plate reader for the fluorescence intensity of ROS and GSH at Ex/Em = 510/610 nm and Ex/EM = 490/520 nm, respectively.

### Determination of MDA levels

The MDA level was determined with an MDA assay (Abcam, ab118970). The retinal tissue was lysed and centrifuged at 13,000 × g and 4 °C for 10 min. The supernatant was taken for the assay. The content of MDA was determined by the sample’s absorbance measured by the OD value at 532 nm under spectrophotometry, according to the steps described in the assay manual.

### Western blotting

Freshly isolated rat retinas were homogenized and lysed in ice-cold radioimmunoprecipitation assay (RIPA) buffer (P0013; Beyotime). The total proteins of the retina were extracted for protein quantitation via a BCA protein assay (Nanjing Senbeijia, BI-WB005). The protein samples were separated on a polyacrylamide-SDS gel and electroblotted onto a nitrocellulose membrane (Bio-Rad, Hercules, CA, USA). After blocking with 5% nonfat milk at room temperature for 1 h, the membranes were incubated with primary antibodies overnight at 4 °C. The primary antibodies were the SLC7A11 antibody (Abmart, 1:1000, TD12509), GPX4 antibody (Abmart, 1:2000, T56959), CD71/TfR polyclonal antibody (Immunoway, 1:500, YT5374), ferritin antibody (Abmart, 1:2000, T55648) and anti-GAPDH antibody (Abcam, 1:2000, ab8245). Then, HRP-conjugated secondary antibodies (Solarbio, 1:5000) were incubated for 60 min at room temperature. Finally, a chemiluminescent reagent mixture was added to the membrane, and the membrane was incubated for 5 min. The membrane was then put into the GelView6000ProII automatic gel imaging system for signal collection and analysis.

### Real-time polymerase chain reaction (PCR) analysis

Total RNA was extracted from the retina tissue lysates with an extraction kit (Omega Bio-Tek, R6934-01). Then, cDNA was generated using a two-step gDNA removal and reverse transcription three-generation master mix (Monad, MR05201). Then, mRNA expression was quantitated using MonAmp ChemoHS qPCR Mix (Monad, MQ00401) by dye assay. The expression of each target gene was normalized to the internal reference GAPDH by the 2^−ΔΔCt^ method, and the relative expression of SLC7A11, GPX4, Transferrin, and FTH was then measured. The procedures of real-time PCR experiments were as follows: 5 min at 95 °C, followed by 40 cycles of 15 s at 95 °C and 32 s at 60 °C. The sequences of primers (designed and provided by Guangzhou Tenng Biotechnology Co., Ltd.) of real-time PCR products are listed in Table 1.

**Table 1:** Primer sequences for real-time PCR.

Gene	Forward primer sequence (5′-3′)	Reverse primer sequence(5′-3′)
SLC7A11	TATGGGACAAGAAACCCAAG	AACCAATTCCTTTAGCCCAT
GPX4	AATGAGGCAAAACCGACGTA	CGGCTGCAAACTCCTTGATT
Transferrin	TCCGAACAACAGAGAGGGAT	CAGATCCTTAGCCCATGCAG
FTH	TGAGCCCTTTGCAACTTC	CCCGGTCAAAATAACAAGAC
GAPDH	CAAGGATACTGAGAGCAAGAGA	AGGCCCCTCCTGTTGTTAT

### Hematoxylin & eosin (HE) staining

The eyeballs removed from the euthanized rats were fixed with 4% paraformaldehyde, embedded in paraffin and made into serial retinal sections parallel to the sagittal axis of the optic nerve. After dewaxing and hydration, the tissue sections were stained with a hematoxylin–eosin staining solution, dehydrated until transparent, and mounted. The retinal histological features and retinal thickness were observed and measured under an optical microscope, and the thickness from the nerve fiber layer to the outer nuclear layer was measured with ImageJ software at a distance of approximately 500 μm from the optic disc. Three sections were taken for each group (unit: μm).

### Transmission electron microscopy (TEM)

The retina tissues were initially fixed in 2.5% glutaraldehyde for 4 h, washed with PBS solutions 3 times, and then underwent secondary fixation with 1% osmium tetroxide for 2 h. The fixed tissues were gradually dehydrated with ethanol and then finally dehydrated with acetone before being embedded in purified embedding solutions overnight at 4 °C. The embedded samples were then placed in a curing oven for polymerization. The samples were then sliced into ultrathin sections ~ 100 nm thick using an ultramicrotome (Leica UC 7). The sections were stained with lead citrate for TEM examination (Tecnai G2 Spirit, FEI Czech Republic s.r.o.). All materials above were supplied by Guangzhou Tenng.

### Immunofluorescence

The eyeballs of the rats were removed as quickly as possible and placed into a special small box (approximately 2 cm in diameter). The tissues were then immersed in an OCT embedding agent and then put steadily into a -80 °C refrigerator. The frozen tissue blocks were placed in a cryostat slicer (KLT-7611, Shenyang Kelaite Innovation Electronic Technology Co., Ltd.) for frozen sections. The tissues were sliced in serial sections of 6 μm and then dried at room temperature for 15 min before being blocked in PBS containing 10% normal goat serum for 1 h at room temperature. After blocking, sections were placed in an immunohistochemical wet box and incubated with primary antibodies RBPMS (Polyclonal, Proteintech, 15,187–1-AP) and GFAP (Polyclonal, Proteintech, 16,825–1-AP) overnight at 4 °C. Sections were rinsed with PBS and incubated with the secondary antibody CoraLite488-conjugated goat anti-rabbit IgG (Proteintech, SA00013-2) for 1 h at room temperature. The slices were sealed in an antifade mounting medium with DAPI and mounted with a clean cover glass. Microscopy images of the tissues were taken under a confocal inverted fluorescence microscope (Nikon C2). The analysis software of the confocal microscope were selected to determine the number of cells and fluorescence intensity per unit area.

### Optical coherence tomography (OCT)

The thickness of the inner retinal layer around the optic disc and of the RNFL was measured via OCT (Heidelberg Engineering, Spectralis OCT). Circle scans for the retinal layer around the optic disc were taken with a 2.5 mm light ring, and the thickness of the RNFL was measured using the average thickness of the four quadrants of the circular RNFL sections: for the right eye, 315°–45° above, 45°–115° on the nasal side, 115°–225° below and 225°–315° on the temporal side; for the left eye, 315°–45° above, 225°–315° on the nasal side, 115°–225° below and 45°–115° on the temporal side.

### Statistical analyses

Quantitative data are summarized as the mean ± standard deviation (SD). The statistical significance of differences among groups was determined by one-way analysis of variance with Tukey’s multiple comparisons test. The statistical analysis was performed on GraphPad version 9.0 (GraphPad Software, San Diego, CA), and a P < value of 0.05 was considered statistically significant.

## Results

### Deferoxamine attenuates visual impairment caused by retinal ischemia‒reperfusion

**Figure 1:**
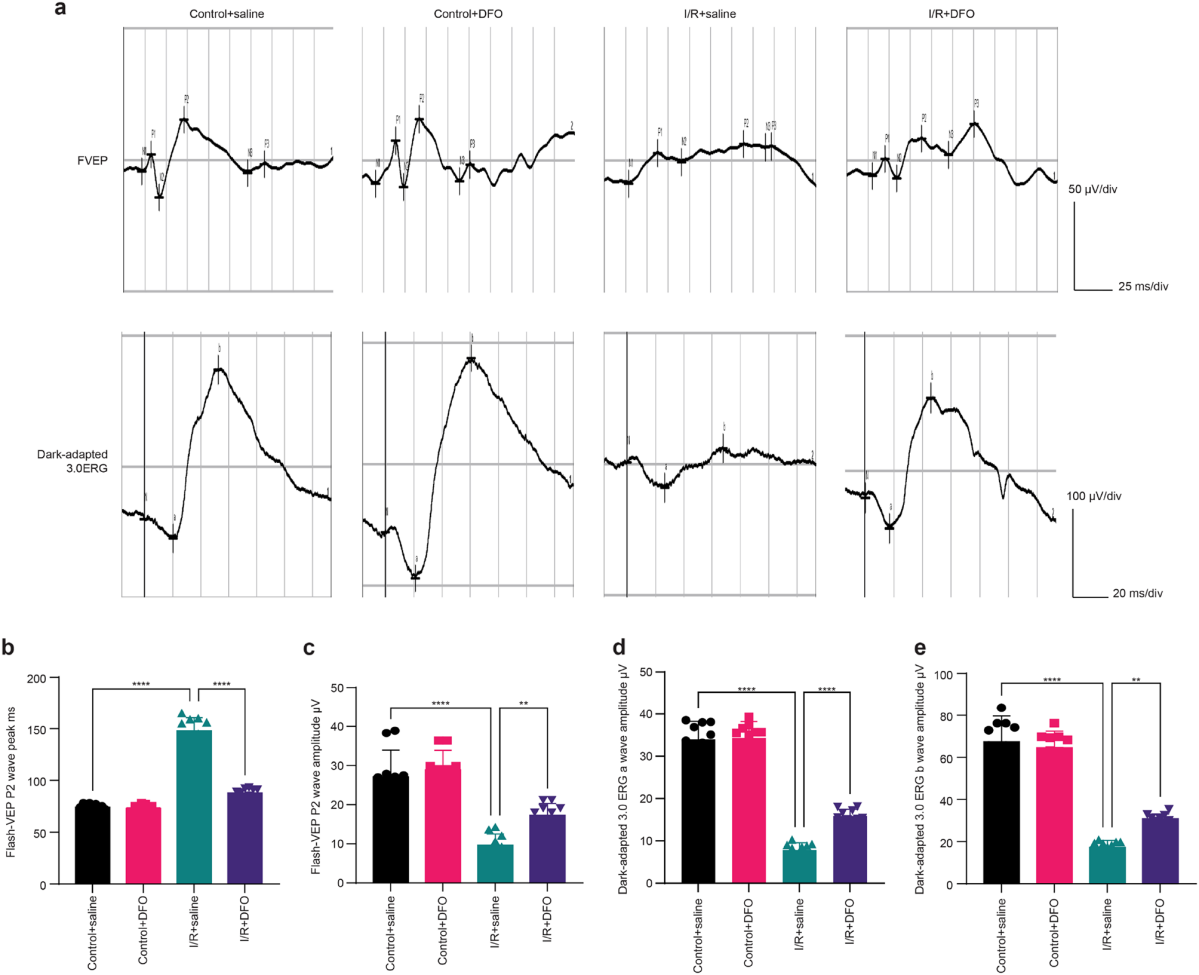
Deferoxamine (DFO) attenuates visual impairment. (**a**) Typical diagrams of FVEP and dark-adaptation 3.0 ERG after 48 h of retinal I/R injuries. (**b, c**) Deferoxamine attenuated the delay in the FVEP P2 peak and the decrease in the P2 wave amplitude (n = 8). (**d, e**) Deferoxamine attenuated the degree of decrease in dark-adaptation 3.0 ERG a-wave and b-wave amplitudes (n = 8). **P < 0.01, ****P < 0.0001 vs. I/R + saline.

Deferoxamine is an iron chelator that can quickly form a stable, nontoxic, water-soluble complex with free or protein-bound ferric iron (Fe^3+^), thereby preventing it from participating in chemical reactions. It has been used clinically for the treatment of iron overload disorders^25^. We performed ERG and VEP tests to evaluate the subject’s retinal visual function after treatment with deferoxamine (Fig. 1a). The ERG results showed heightened amplitudes of a & b waves in the deferoxamine arm’s dark-adaptation 3.0 potentials (Fig. 1d, e). The VEP results showed shorter latency and heightened amplitudes in the P2 waves of the deferoxamine arm (Fig. 1b, c).

### Ferroptosis is involved in retinal ischemia‒reperfusion

**Figure 2:**
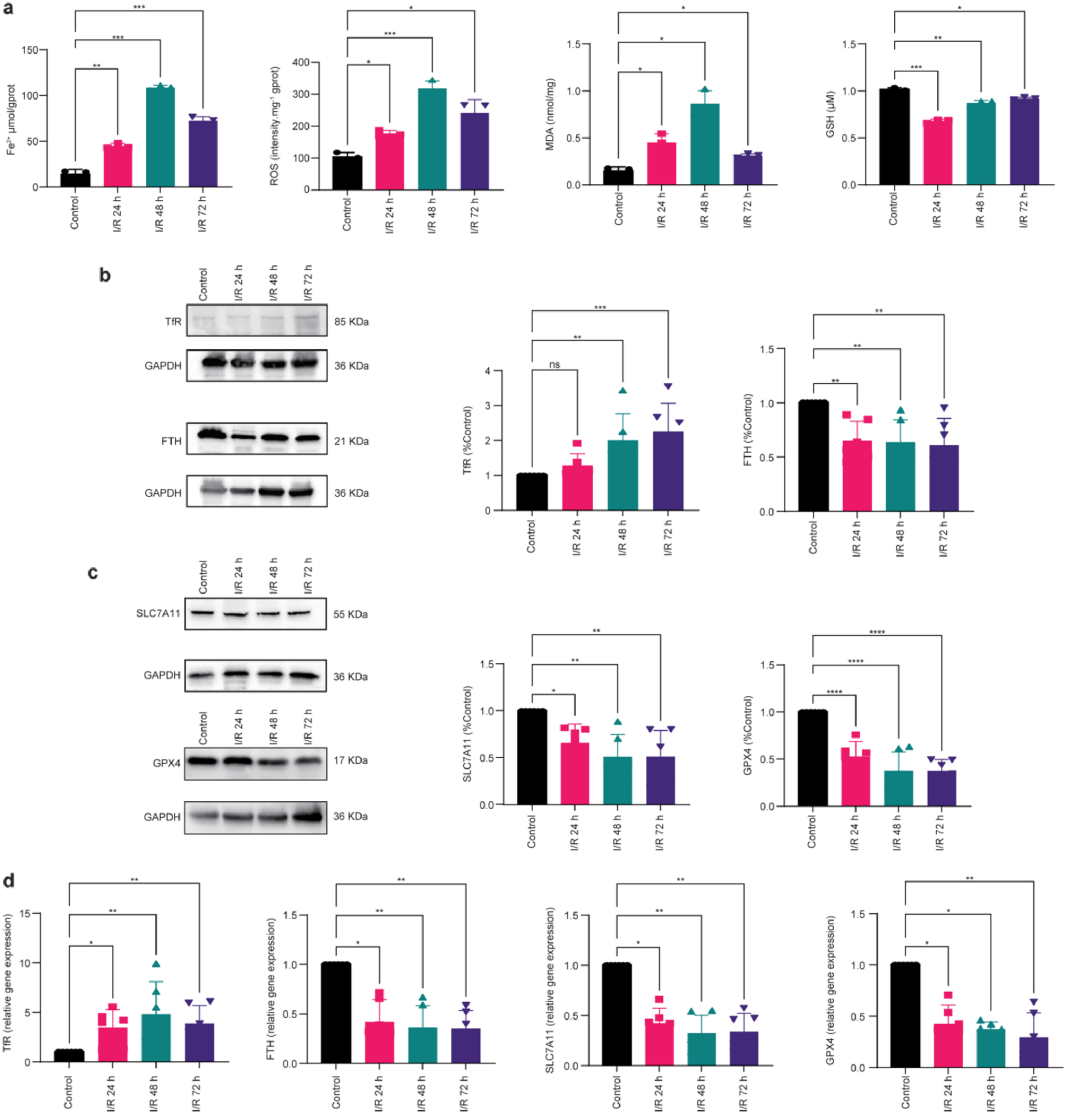
Ferroptosis is involved in retinal I/R injury. Within 3 days of retinal I/R injury: (**a**) The contents of Fe^2+^, ROS, and MDA increased, and the content of GSH decreased (n = 3). (**b**) WB tests showed that the expression of TfR increased and the expression of FTH decreased (n = 6). (**c**) WB tests showed that the expression of SLC7A11 and GPX4 decreased (n = 6). (**d**) Real-time PCR showed that TfR mRNA expression levels increased and FTH, SLC7A11, and GPX4 mRNA expression levels decreased (n = 6). ns: not significant, *P < 0.05, **P < 0.01, ***P < 0.001, ****P < 0.0001 vs. control.

We performed an in vivo study to validate the presence of ferroptosis in the early phase after retinal I/R injury. We demonstrated this by evaluating the markers of oxidative stress and changes in key genes and proteins during iron metabolism, such as TfR, FTH, SLC7A11, and GPX4^8^. Within 3 days after the retinal I/R injuries, compared with the control group, the levels of Fe^2+^, ROS and MDA rapidly increased after the I/R injuries in the experimental group and peaked at 48 h. Although these indicators recovered by 72 h, the levels of Fe^2+^, ROS, and MDA in the experimental group remained higher at all time points than those in the control group (Fig. 2a). Compared with the control group, the GSH levels in the retinas of the experimental group decreased rapidly after I/R injury, dropped to the lowest value at 48 h, started to recover by 72 h and remained lower than those of the normal group at all time points (Fig. 2a). In the experimental group, the expression of the TfR protein and the corresponding mRNA increased over time after injury, while the expression of FTH, SLC7A11, and GPX4 proteins and the corresponding mRNA decreased over time (Fig. 2b, c, d).

### Ferroptosis occurs in various cell types in retinal ischemia‒reperfusion

**Figure 3:**
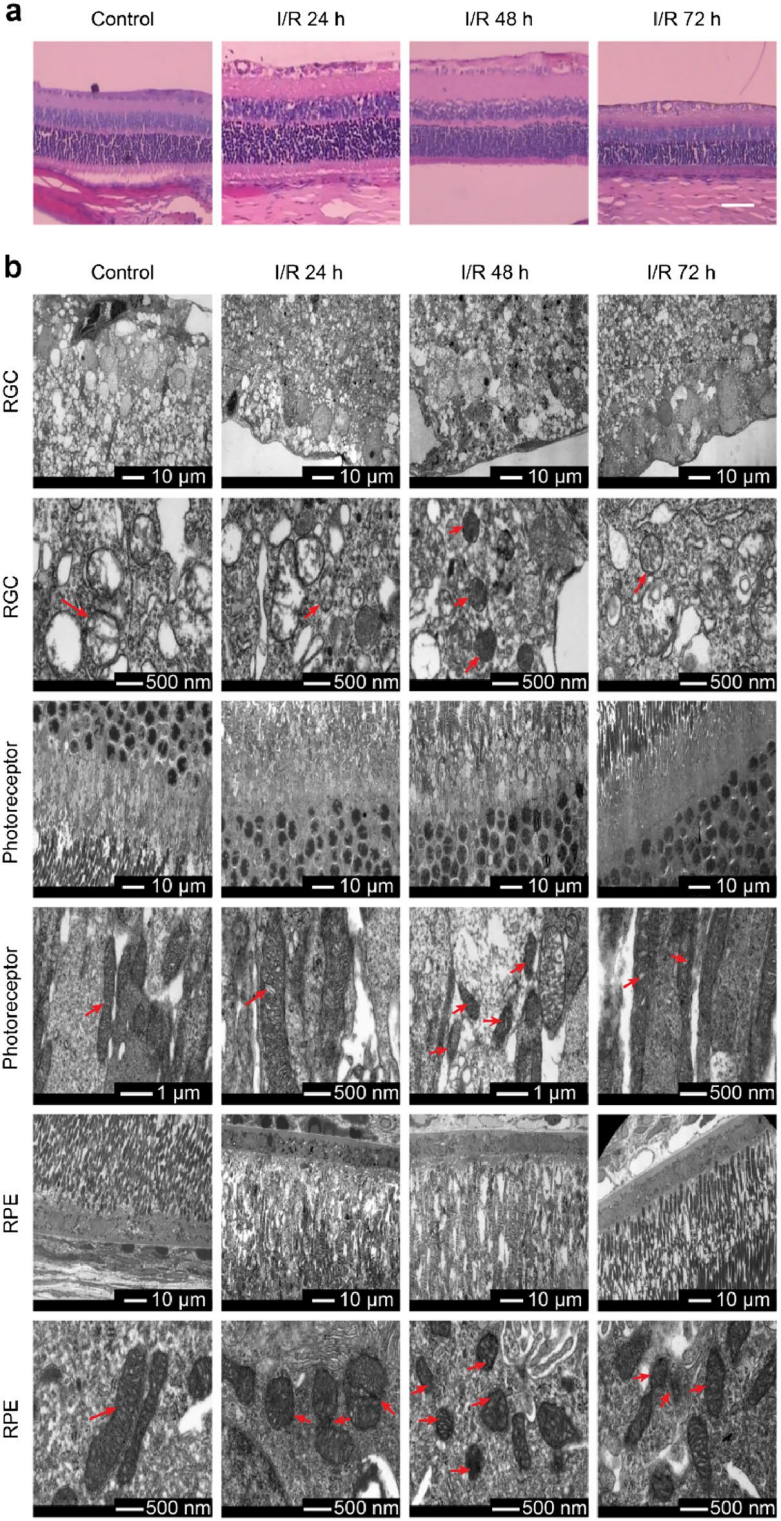
Ferroptosis occurs in various cell types in retinal I/R injuries. Within 3 days of retinal I/R injury: (**a**) HE staining indicated retinal thickening, vacuole signs in RGCs, and decreased RGC cell counts (n = 3). Scale bar = 50 µm. (**b**) TEM observations showed shrinkage of mitochondria in RGCs, photoreceptor cells, and RPE cells (n = 3, the red arrow represents mitochondria). Scale bar = 10 µm/500 nm.

HE staining was used to observe the pathological changes in the retina after retinal I/R injury. In the control group, the rat’s retinal structure was compact, and the arrangement of RGCs was orderly. In the experimental group, a large number of vacuoles appeared in the retinal nerve fiber layer and ganglion cell layer after I/R within 24 h, and the thickness of the retina increased. By 48 h, the edema volume peaked, and the inner and outer ganglia were swollen, loose, and vacuolated. By 72 h, the nuclei of the ganglion cell layer became compressed, the cell counts decreased, retinal edema subsided, and the retina thickness recovered (Fig. 3a). We then conducted further morphological examinations of the changes in the mitochondria in retinal cells via TEM. The retinal RGC mitochondria of the control rats appeared to be larger and in normal spherical shapes, while the mitochondria of RPE and photoreceptor cells were long and rod-shaped, with smooth outer membranes and dense crista lining the inter membranes. In the experimental group, the arrangement of the cone and rod cell layers was disorderly, and the mitochondria in the RPE, photoreceptor cells, and RGC cells tended to be visibly smaller. The mitochondria of the experimental group had already shown signs of shrinkage within 24 h and became more obvious by 48 h. By 72 h, mitochondrial shrinkage had recovered, and the volume of mitochondria in each layer started to recover and became closer to that of the control group (Fig. 3b).

### Deferoxamine inhibits ferroptosis

**Figure 4:**
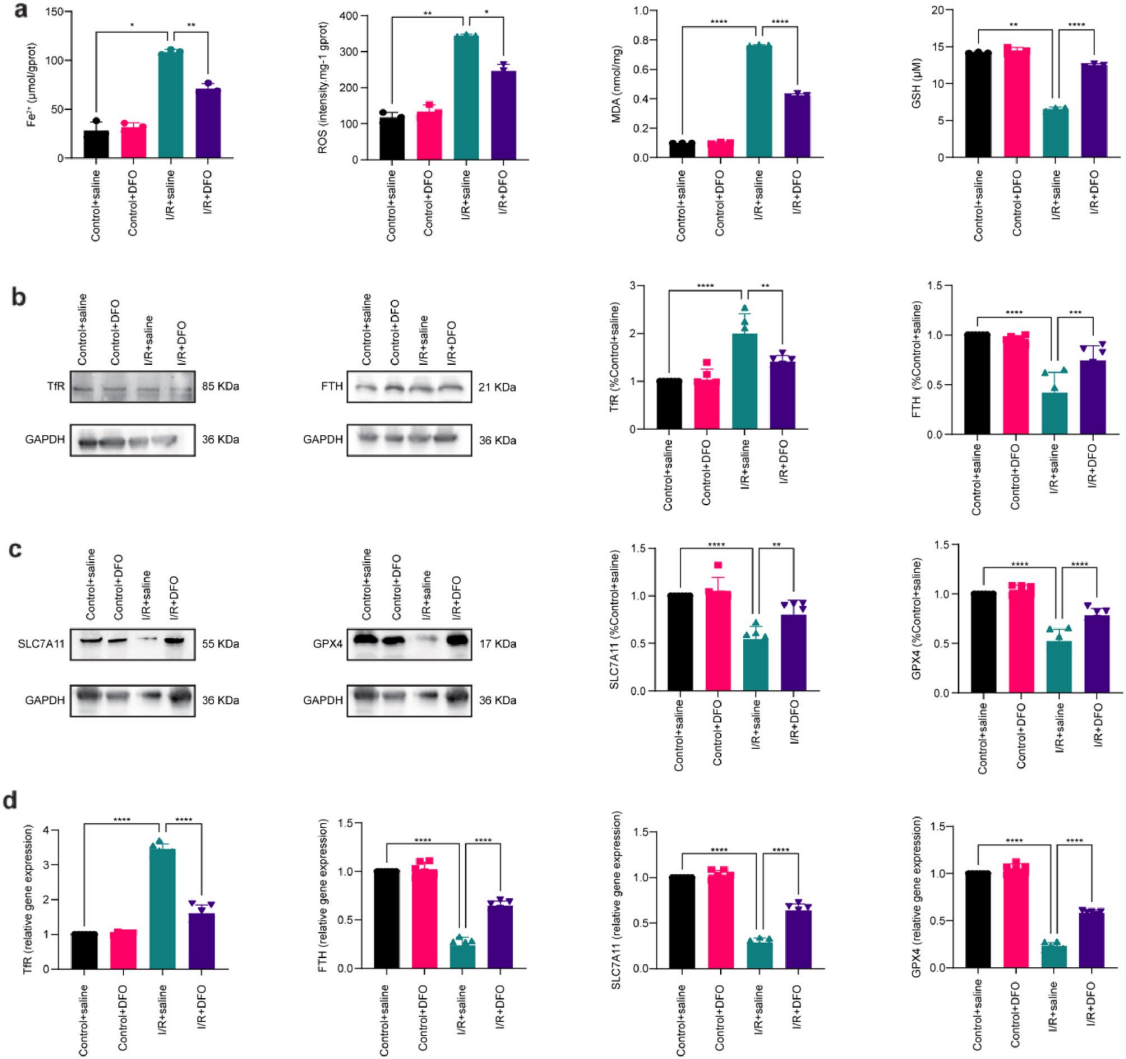
Deferoxamine inhibits ferroptosis. (**a**) Deferoxamine attenuated the increase in retinal iron, ROS, and MDA levels and the decrease in GSH levels (n = 3). (**b**) WB tests showed that deferoxamine inhibited the expression of TfR and increased the expression of FTH (n = 6). (**c**) WB tests showed that deferoxamine increased the expression of SLC7A11 and GPX4 (n = 6). (**d**) Real-time PCR showed that deferoxamine restored the levels of TfR, FTH, SLC7A11, and GPX4 mRNA (n = 6). *P < 0.05, **P < 0.01, ***P < 0.001, ****P < 0.0001 vs. I/R + saline.

By assessing the effect of deferoxamine on key factors in iron metabolism after retinal I/R injury, we found that deferoxamine significantly attenuated the accumulation of Fe^2+^ in the retina, reduced the overproduction of ROS and MDA, and increased the level of GSH after I/R injury (Fig. 4a). Additionally, within the model group, the expression of the TfR protein and the corresponding mRNA decreased in the deferoxamine arm, and the expression of FTH, SLC7A11, and GPX4 proteins and the corresponding mRNA increased in the deferoxamine arm. This evidence indicates that deferoxamine can inhibit ferroptosis after retinal I/R injury (Fig. 4b, c, d).

### Deferoxamine attenuates retinal edema in the early phase after retinal I/R and inhibits RGC death and Müller cell proliferation

**Figure 5:**
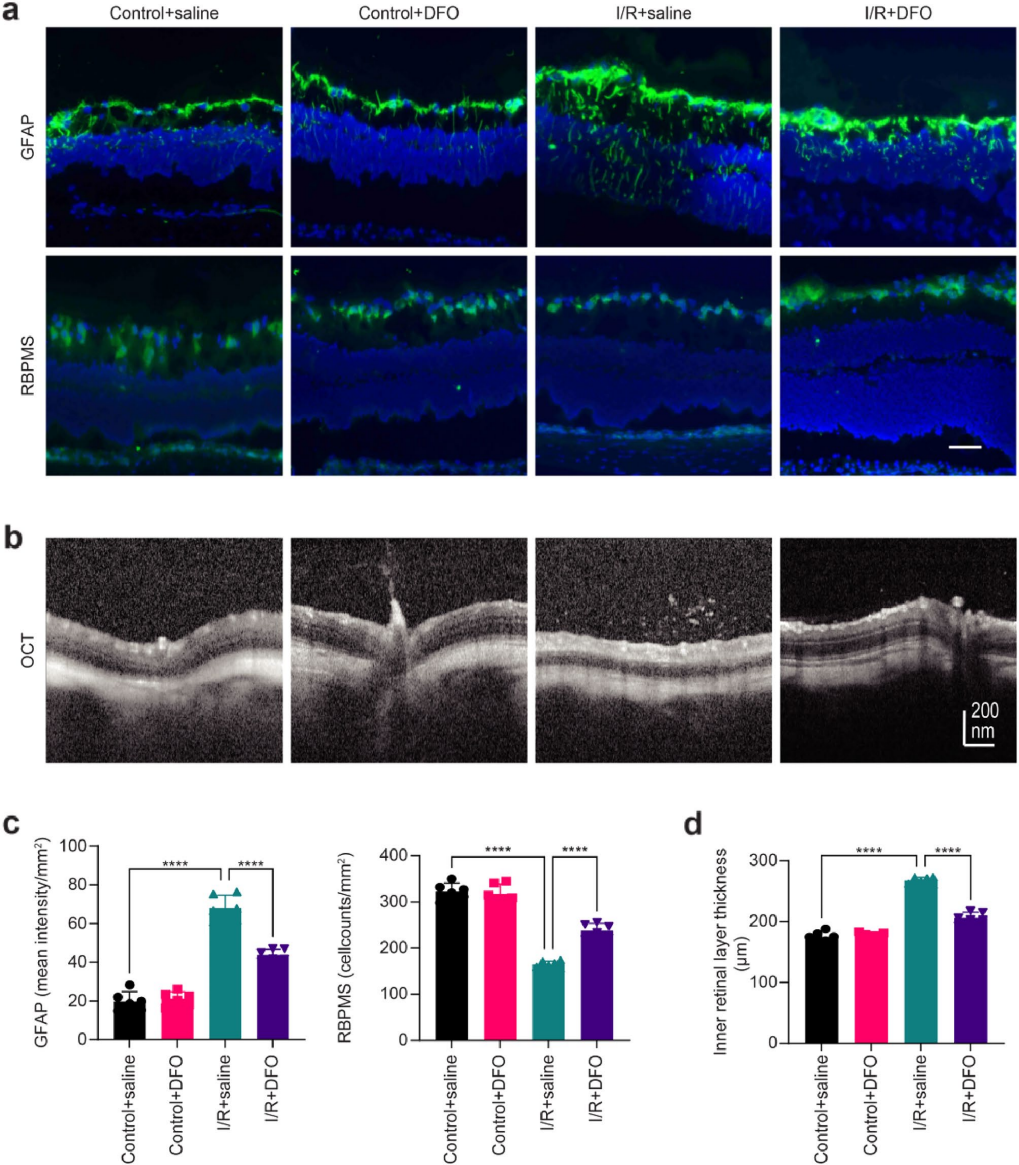
Deferoxamine attenuates retinal edema in the early stage after retinal I/R injury and inhibits RGC death and Müller cell proliferation. (**a**) Representative immunofluorescence images showed increased expression of GFAP (green) and decreased expression of RBPMS (green) in RGCs after I/R, and these effects were inhibited by DFO (n = 3). Blue: DAPI. Scale bar = 50 µm. (**b**) Typical diagrams of OCT after 48 h of retinal I/R injuries. (**c**) Quantitative analysis of the immunofluorescence intensity of GFAP and RBPMS (n = 3). (**d**) Deferoxamine significantly reduced retinal edema, which is likely to appear in the early stage after retinal I/R injury (n = 6). ***P < 0.001, ****P < 0.0001 vs. I/R + saline.

Retinal I/R injury is a result of multiple contributing factors. Its main clinical signs include neuron death and neural inflammation^19^. Previous studies have found that the retina tends to show signs of severe edema in the early stage after an I/R injury, with the ganglion cells vacuolated, cell counts decreased and the cell distribution becoming disorderly^26^. At this stage, the Müller cells become dedifferentiated through glial reaction, manifested as glial cell hypertrophy and glial fiber acidic protein (GFAP) overexpression^26^. The retinal ganglion cell marker RBPMS and the Müller cell marker GFAP were detected through immunofluorescence assays on frozen retinal sections. We found that GFAP expression levels increased and RBPMS expression levels decreased after I/R injury. We found that the RGC count increased, the expression level of RBPMS increased, the abnormal proliferation of Müller cells was alleviated, and the expression level of GFAP decreased (Fig. 5a, b). We also evaluated the retinal structure by OCT measurement of retinal thickness in the early stage after retinal I/R injury. The retina was highly edematous at 48 h after injury. Deferoxamine significantly reduced retinal edema, which is likely to appear in the early stage after retinal I/R injury (Fig. 5c, d).

## Discussion

Retinal injuries caused by ischemia‒reperfusion are associated with RGC damage, changes to retinal morphologies, and loss of retinal functions, which eventually leads to loss of vision^1,2,23^. In this study, we demonstrated through an in vivo experiment that ferroptosis was involved in retinal ischemia‒reperfusion and that deferoxamine may inhibit ferroptosis by reducing iron overload and ROS accumulation in retinal tissues and therefore improve the cell’s resilience to oxidative stress, which may contribute to the retention of retinal structures and functions. This finding suggests that ferroptosis caused by iron overload may be an effective therapeutic target for retinal ischemia‒reperfusion injury, which promises a new approach for the clinical treatment of ischemic eye diseases.

After the completion of the deferoxamine treatment, we followed up with retinal functional examinations: we performed FVEP, which reflects the function of a large range of retinal and optic nerve pathways, and dark adaptation 3.0 ERG, which evaluates the functionality of the retina. These are two of the most recognized efficacy indicators for eye disease treatment. The results showed that deferoxamine can alleviate P2 latency and the degree of amplitude decrease in FVEP and can also reduce the amplitude decrease of the a- and b-waves on dark-adapted 3.0 ERGs. These findings suggested that deferoxamine may attenuate visual impairment caused by retinal ischemia‒reperfusion.

The remainder of this section will elaborate on the mechanism underlying deferoxamine’s therapeutic effect on retinal I/R injury-related visual damage. Ferroptosis is associated with four essential factors: iron overload, free radical production, accumulation of lipid peroxides, and reduction in antioxidants^17^. Iron overload refers to the pathological phenomenon in which an excessive level of iron accumulation in the organism causes cell damage and organic disorders^27^. The metabolism of intracellular iron is regulated by various factors, including the ferric complex (Tf-Fe^3+^) formed by the combination of extracellular Fe^3+^ and transferrin. Fe^3+^ enters the cell through the ferric complex by binding to the TfR1 receptor on the cell membrane. When Fe^3+^ enters the cell, it is reduced to Fe^2+^, and excess iron ions are stored in ferritin^28^. Thus, both increased iron absorption and reduced iron storage will lead to an increase in iron content in the cell, which triggers downstream signaling cascades that promote ferroptosis. The iron level in the cell may increase as a result of the increase in iron absorption mediated by transferrin through the transferrin receptor (TFRC) pathway or the degradation of ferritin through autophagy, both of which can promote ferroptosis^25^. Ferritin is composed of the heavy chain (FTH) and the light chain (FTL). Previous research has suggested that FTH is the functional unit maintaining the iron absorption function of ferritin^29^. Therefore, in this study, we chose the Fe^2+^ level in the retina and the TfR and FTH protein and gene expression levels after I/R injury. We found that at different time points after I/R injury, the Fe^2+^ content and the expression level of TfR mRNA and protein increased, and the expression level of FTH mRNA and protein decreased in a time-dependent manner. These results indicated that the increase in transferrin expression and the degradation of ferritin in the early stage after retinal ischemia‒reperfusion could lead to an increase in free iron ion content in retinal tissues, thereby inducing ferroptosis in cells.

Excessive Fe^2+^ can induce the Fenton reaction and produce active oxygen, which greatly accelerates the lipid peroxidation of unsaturated fatty acids in organisms. When the oxidation reaction caused by iron deposition exceeds the antioxidant capacity of cells, it can cause the initiation of oxidation stress signals, which directly or indirectly cause damage to cellular macromolecules such as proteins, nucleic acids, and lipids, leading to cell damage or death^10^. We examined the ROS and MDA content in the animal models and found that after I/R injury, the levels of ROS and MDA increased significantly, suggesting that the level of oxidative stress in the retina increased significantly after injury.

The System Xc^−^-GSH-GPX4 pathway is an important antioxidant pathway regulating ferroptosis in cells. System xc^−^ is composed of two subunits called SLC7A11 and SLC3A22, where SLC7A11 is the main functional subunit for transportation. It exchanges extracellular cystine, the raw material involved in GSH synthesis, with intracellular glutamic acid in a 1:1 ratio. GSH, as an important antioxidant, removes free radicals in organisms. GSH is also a reducing cofactor of glutathione peroxidase (GPX4), which uses two GSH molecules as electron donors to reduce phospholipid hydroperoxide (PL-OOH) to nontoxic lipid alcohol (L-OH) and thereby protects the cell membrane structure and functionality. When the System Xc^−^-GSH-GPX4 pathway is inhibited, the antioxidant capacity of cells is reduced, thereby promoting ferroptosis^6,7,9,10,30^. In our experiment, we found that the content of GSH and the expression levels of SLC7A11 and GPX4 proteins and their mRNA dropped in the damaged retinas. In addition, we found typical structural signs of ferroptosis, i.e., mitochondrial shrinkage and a decrease in mitochondrial cristae, across multiple cell types, including RGCs, photoreceptor cells, and pigment epithelial cells, via TEM examinations. This evidence indicates that ferroptosis occurs in a range of cell types in retinal ischemia‒reperfusion.

Studies have demonstrated the role and mechanism of deferoxamine in the treatment of neurodegenerative diseases and neurovascular diseases and the promotion of the healing of diabetes-associated wounds^31–33^. At the same time, deferoxamine has also been suggested for managing obesity due to its excellent antioxidant properties by alleviating oxidative stress and inflammation^34^. In the present study, the rats in the deferoxamine arm showed a significant reduction in the content of Fe^2+^ and the expression of TfR and an increase in the expression of FTH, which illustrated that deferoxamine can effectively combine with ferric iron to reduce the iron load of the body, thereby reducing the body’s homeostatic response to alleviate iron level stress. In addition, deferoxamine also reduces oxidative stress in tissues by reducing the content of ROS and MDA and increasing the content of GSH and the expression of SLC7A11 and GPX4, thereby preventing cell damage.

In the experiment, we observed clear structures and orderly arrangement of the cells in the retinal layers in the normal rats by HE staining and immunofluorescence examinations. In the model rats with I/R injuries, we found serious retinal edema soon after the injury, and the RGCs became loosely distributed and vacuolated and decreased in cell number. When the deferoxamine treatment was complete, the arrangement of the retinal cells returned to a nearly orderly state, and the number of RGCs increased with fewer signs of vacuoles. GFAP is a biomarker of Müller cell activation. Müller cells are a type of glial cell that runs through all layers of the retina. Neuroinflammation caused by the glial reaction initiated by Müller cells plays a key role in the pathogenesis of retinal ischemia‒reperfusion. The activation of Müller cells induces proliferation and morphological changes in retinal cells and changes the expression levels of certain retinal enzymes and receptors. Deferoxamine delivery reduces nerve damage and inflammation by reducing the death of RGCs and the abnormal proliferation of Müller cells. OCT is a good index to evaluate the thickness of the whole retina and RNFL. In our experiment, the OCT examination showed that the whole retina and the RNFL were severely edematous in the early stage after I/R injury, with the boundaries between layers becoming indistinguishable. Only the thickness of the inner retina was measurable at this state. Deferoxamine delivery, however, has been shown to greatly reduce retinal edema.

However, our research is limited to animal experiments, and the specific molecular mechanism of ferroptosis calls for further investigations at the cellular level. Moreover, our research is limited to the early stage after retinal ischemia‒reperfusion injury, and whether ferroptosis is involved in the middle and late stages after retinal ischemia‒reperfusion injury remains unclear and awaits further exploration.

In conclusion, this study found that retinal ischemia‒reperfusion can induce ferroptosis in a variety of retinal cells. Deferoxamine protects the structural and functional soundness of the retina by salvaging retinal ganglion cells from cell death and inflammatory reactions. This is achieved by inhibiting ferroptosis through the simultaneous inhibition of hemochromatosis, the initiation of transferrin, and the degradation of ferritin. These findings indicate that ferroptosis inhibition could be a new therapeutic approach for treating ischemic retinal diseases.
